# Prediction of the Ki-67 marker index in hepatocellular carcinoma based on Dynamic Contrast-Enhanced Ultrasonography with Sonazoid

**DOI:** 10.1186/s13244-022-01320-6

**Published:** 2022-12-20

**Authors:** Zhe Huang, PingPing Zhou, ShanShan Li, Kaiyan Li

**Affiliations:** Department of Medical Ultrasound, Tongji Hospital, Tongji Medical College, Huazhong University of Science and Technology, Wuhan, China

**Keywords:** Contrast-enhanced ultrasound, Quantitative perfusion, Carcinoma (Hepatocellular), Ki-67 Antigen, Prognosis

## Abstract

**Background:**

Ki-67 is widely used as a proliferative and prognostic factor in HCC. This study aimed to analyze the relationship between dynamic contrast-enhanced ultrasonography (DCE-US) parameters and Ki-67 expression.

**Methods:**

One hundred and twenty patients with histopathologically confirmed HCC who underwent DCE-US were included in this prospective study. Patients were classified according to the Ki-67 marker index into low Ki-67 (< 10%) (*n* = 84) and high Ki-67 (≥ 10%) groups (*n* = 36). Quantitative perfusion parameters were obtained and analyzed.

**Results:**

Clinicopathological features (pathological grade and microvascular invasion) were significantly different between the high and low Ki-67 expression groups (*p* = 0.029 and *p* = 0.020, respectively). In the high Ki-67 expression group, the peak energy (PE) in the arterial phase and fall time (FT) were significantly different between the HCC lesions and distal liver parenchyma (*p* = 0.016 and *p* = 0.025, respectively). PE in the Kupffer phase was significantly different between the HCC lesions and the distal liver parenchyma in the low Ki-67 expression group (*p* = 0.029). The difference in PE in the Kupffer phase between HCC lesions and distal liver parenchyma was significantly different between the high and low Ki-67 expression groups (*p* = 0.045). The difference in PE in the Kupffer phase between HCC lesions and distal liver parenchyma < − 4.0 × 10^7^ a.u. may contribute to a more accurate diagnosis of the high Ki-67 expression group, and the sensitivity and specificity were 82.9% and 38.7%, respectively.

**Conclusions:**

The DCE-US parameters have potential as biomarkers for predicting Ki-67 expression in patients with HCC.

**Key points**

1. High Ki-67 expression of HCC indicated the potential deterioration of HCC, as presented by poor histological differentiation and more microvascular invasion.
2. High Ki-67 expression HCC group had lower difference in peak energy in the Kupffer phase.
3. The difference in peak energy in the Kupffer phase may contribute to diagnosis of the high Ki-67 expression HCC group.

## Introduction

Hepatocellular carcinoma (HCC) is the sixth most common cause of cancer-related deaths [1]. Ki-67 protein in the nucleus is associated with cell proliferation, which may indicate the aggressiveness of tumors [2]. Paraffin-embedded section immunohistochemistry is used to obtain the Ki-67 marker index, and the positive rate could be used as an indicator for the prognosis of patients [2]. Previous studies have shown that higher Ki-67 LI confers a fast progression and poor prognosis for HCC patients [2–8]. Ki-67 is considered a novel treatment target for HCC because it is highly expressed in most malignant cells but is rarely detected in normal cells [9]. The identification of high and low levels of Ki-67 expression is critical for prognosis and for making treatment decisions. However, it is difficult to distinguish subtle differences between HCC with different Ki-67 expression levels by conventional imaging.

Contrast-enhanced ultrasound (CEUS) has been widely used to evaluate microcirculation perfusion in HCC because of its unique advantages of real-time scanning, non-radiation, reproducibility, and convenience. Sonazoid (GE Healthcare, Waukesha, WI, USA) is a second-generation ultrasound contrast agent. Sonazoid-CEUS is currently one of the most commonly used methods to diagnose liver tumors, especially HCC [10]. SonoVue attenuates rapidly, especially in patients with liver cirrhosis with poor liver perfusion, the capacity for detection and diagnosis is limited. Sonazoid has high affinity with Kupffer cells in the liver reticuloendothelial system, making parenchyma-specific imaging possible and allowing enough time for scanning the whole liver [11, 12]. The unique Kupffer stage of Sonazoid CEUS has been shown to correlate with the histological grade of HCC compared with other ultrasound contrast agents [13].

Dynamic contrast-enhanced ultrasound (DCE-US) is a quantitative imaging technique based on conventional CEUS that can be used to precisely quantify tissue perfusion and monitor the efficacy of anti-angiogenic and anti-inflammatory treatments [14, 15]. DCE-US can be used to understand the tissue perfusion and angiogenesis of lesions by quantitatively analyzing the dynamic flushing and CEUS flushing processes. However, no exhaustive study of DCE-US-Sonazoid for predicting Ki-67 expression before surgery has been conducted.

This study aimed to analyze the relationship between DCE-US parameters and Ki-67 expression.

## Materials and methods

### Patients

**Fig. 1:**
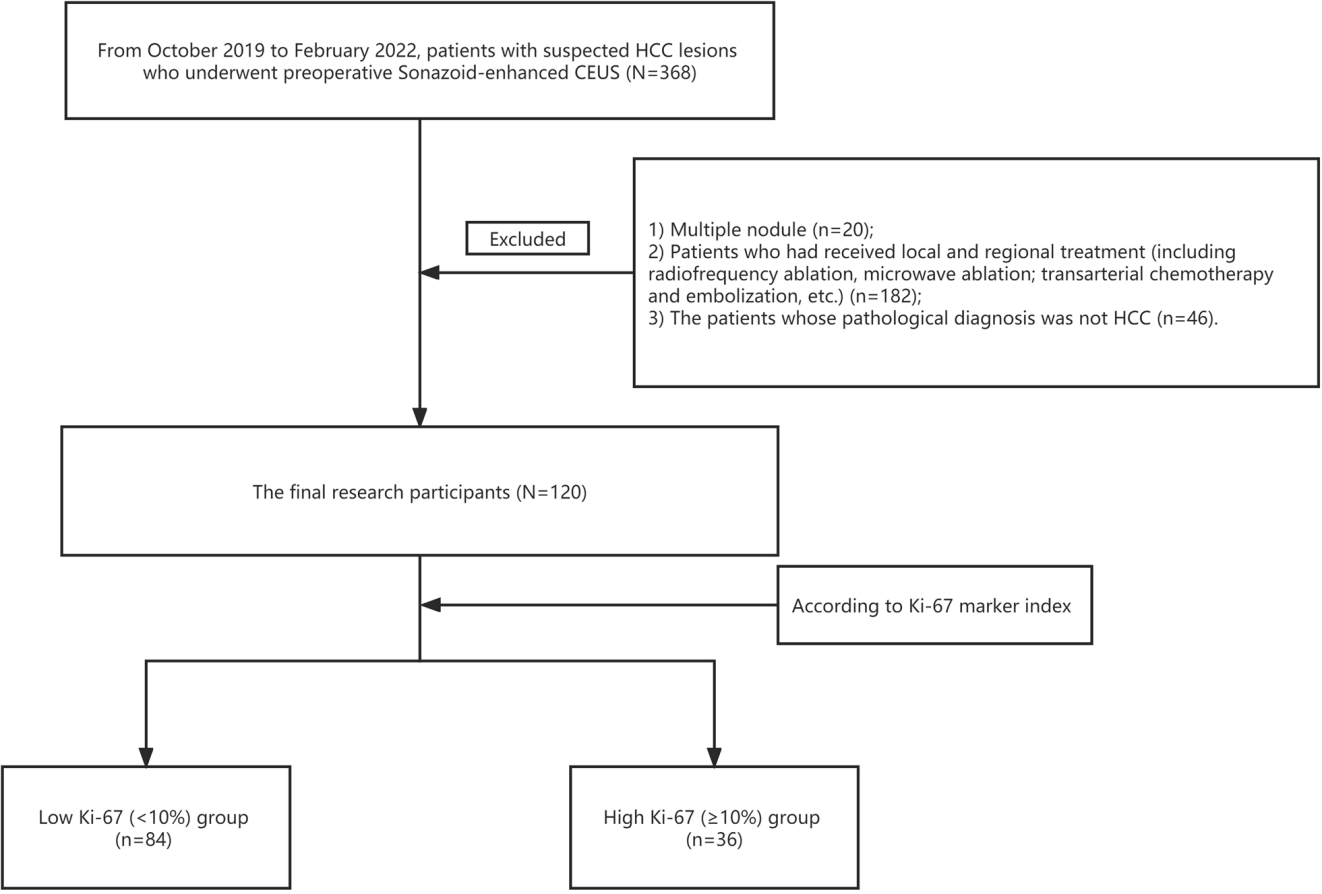
Flowchart for patient selection

Our study was approved by the institutional review board of the hospital. It was conducted in accordance with the principles of the Declaration of Helsinki. For each participant, written informed consent was obtained before US examination. Patients with suspected HCC lesions were prospectively enrolled between October 2019 and February 2022. Patients underwent Sonazoid-CEUS within 1 week before surgical resection. The exclusion criteria were as follows: (1) multiple nodules (i.e., > 5 nodules); (2) patients who had received locoregional treatment (e.g., microwave ablation and embolization); and (3) patients whose pathological diagnosis was not HCC (Fig. 1).

### Immunohistochemical staining, analysis

All patients with hepatocellular carcinoma underwent histological evaluation after hepatectomy. Specimens were evaluated by a pathologist blinded to clinicopathological features and CEUS data. After surgery, the specimens were fixed in 10% formaldehyde solution and embedded in paraffin. Sections were examined microscopically after staining with hematoxylin and eosin (H & E) and Azan-Mallory, and reticulin impregnation. Cell differentiation grade was classified according to Edmonson-Steiner classification. Four-micron-thick serial sections were cut from paraffin-embedded blocks and immunohistochemically analyzed for Ki-67 expression. Tumor tissue sections were stained with monoclonal mouse anti-human Ki-67 antibody. Ki-67 expression was assessed by counting the frequency of 1 Ki-67 positive cell. Ki-67 was considered positive when the nuclei were stained brownish yellow. Immunoreactive cells from 1000 malignant cells were used to determine percentiles of Ki-67-positive tumor cells and to score areas in the tumor with the highest number of positive nuclei (hot spots). The results of Ki-67 IHC were classified as continuous data from 0 to 100%, and according to previous studies, immune-reactive cells were classified as either low Ki-67 expressing (≤ 10% immunoreactivity) or high Ki-67 expressing (> 10% immunoreactivity) [16–18].

### Examination procedure of Sonazoid-CEUS

All the patients underwent CEUS using a Logic E9 US system (GE Healthcare, Wauwatosa, WI) with a convex array probe. The frequency of the transducers ranged from 2 to 5 MHz. All ultrasound examinations were performed by one experienced radiologists with 30 years of clinical experience with grayscale ultrasound and more than 10 years of clinical experience with CEUS. The patients were required to hold their breath or take shallow breaths to obtain a good and stable imaging plane. Before CEUS, each patient was thoroughly examined with conventional and color Doppler ultrasound. Thereafter, the CEUS examination was performed with an intravenous injection of a single bolus of 0.5 ml Sonazoid contrast agent, which was then rinsed with 10 mL of 0.9% saline via an antecubital vein. The CEUS operating mode and a chronograph were initiated simultaneously when the contrast agent was administered. Four-phase continuous CEUS observations were performed, including arterial (0–30 s), portal vein (31–120 s), late (121–360 s) and Kupffer phases. Kupffer phase was defined starting from 10 min later after injection of Sonazoid. Continuous imaging was performed immediately after injection of the contrast agent and lasted for 15 min.

### Quantitative analysis of CEUS

**Fig. 2:**
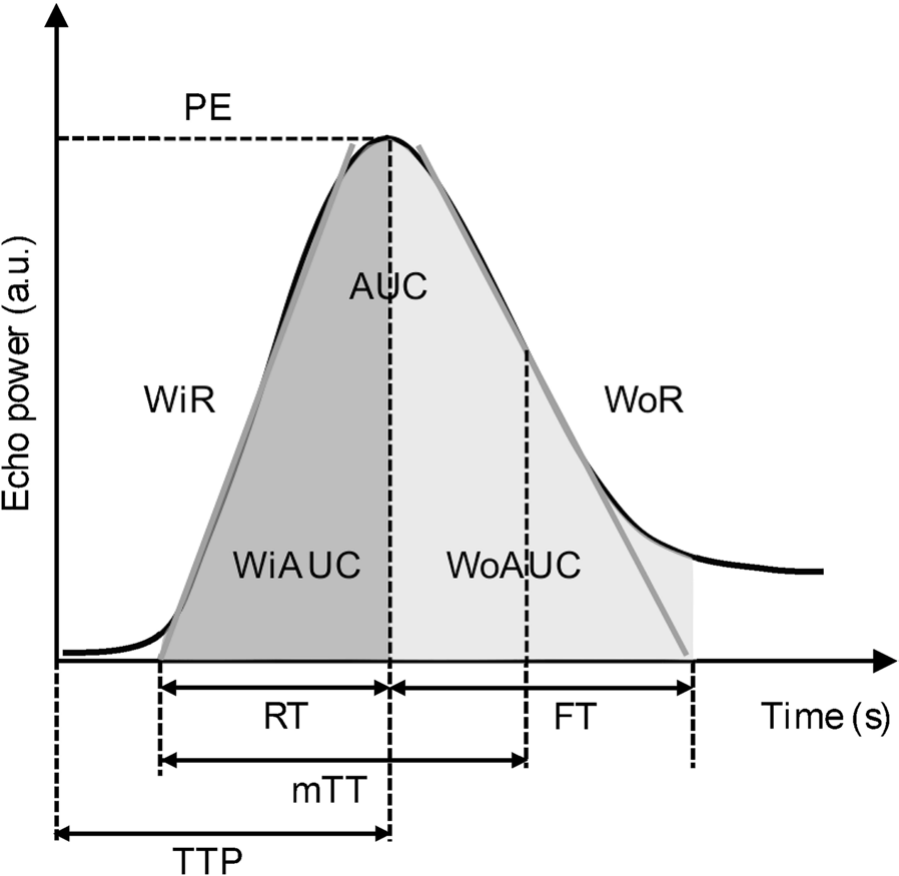
The perfusion parameters obtained from the quantitative analysis of the contrast-enhanced ultrasound examination. CAT, contrast agent arrival time; PE, peak energy; TTP, time to peak; RT, rise time; WiR, wash-in area under the curve; WiAUC, wash-in area under the curve; HDT, half decrease time; FT, fall time; WoR, wash-out area under the curve; WoAUC, wash-out area under the curve; mTT, mean transit time; WiWoAUC, WiWoAUC + WiAUC

**Fig. 3:**
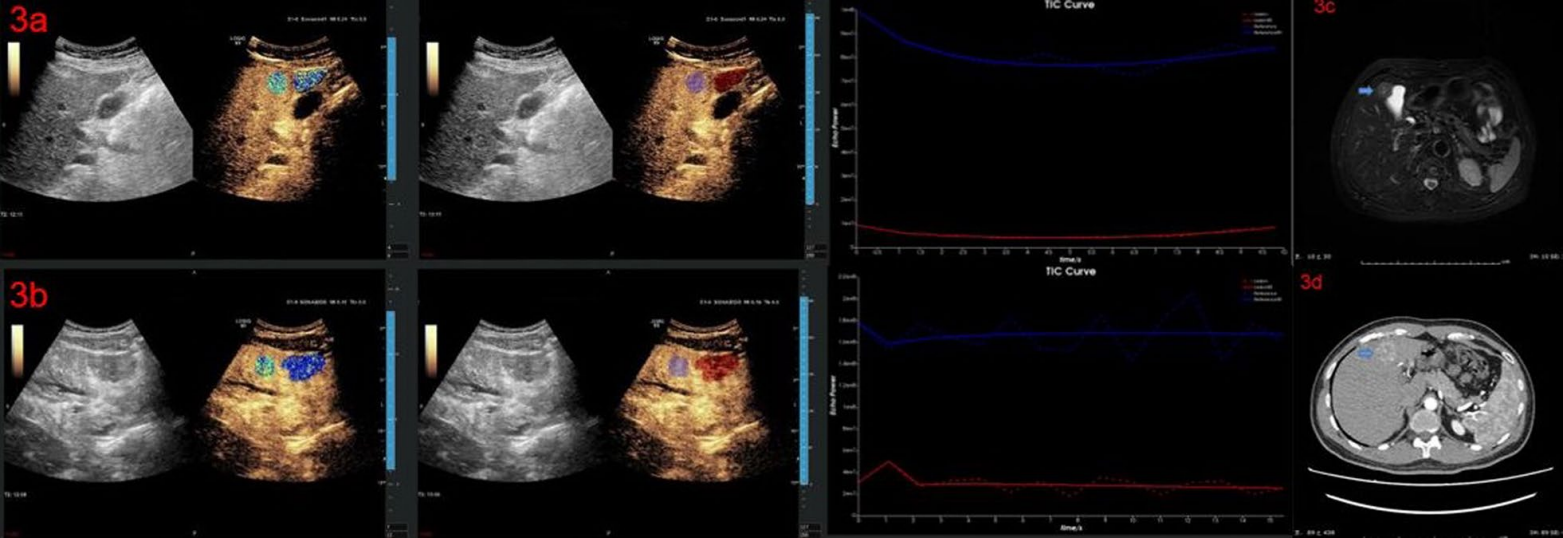
Dynamic contrast-enhanced ultrasound perfusion analysis using the NovoUltrasound Kit (NUK, version 1.5.0, GE Healthcare Shanghai) of histopathological proven hepatocellular carcinoma. **3a** PE in the Kupffer phase of high Ki-67 expression group; **3b** PE in the Kupffer phase of low Ki-67 expression group; **3c** MRI T2 image of 3a; **3d** The CT arterial phase of 3b

The results of CEUS were analyzed using quantitative analysis software (NovoUltrasound Kit (NUK, version 1.5.0, GE Healthcare Shanghai)), and the operator was not informed of the histopathological diagnosis. Analysis were performed by one abdominal radiologists with 5 years of experience. A region of interest was placed in the HCC lesion and distal liver parenchyma. The entire lesion was included in the region of interest of HCC lesion. In addition, we selected a reference region (of any size) within the distal normal-appearing liver tissue in the same depth from the transducer as the target lesion. Motion compensation is used to reduce breathing motion artifacts. A time–intensity curve (TIC) was then generated, reflecting the transition of the ultrasound contrast agent. The CEUS quantitative parameters were extracted as follows: contrast agent arrival time (time from 0 to start of enhancement), peak energy (PE; maximum enhancement intensity), time to peak (the time required to reach PE of arterial phase after contrast agent injection), rise time (the time it takes for the intensity of the contrast agent to go from 5 to 95% PE of arterial phase), wash-in area under the curve (the maximum slope of the TIC, expressed as a tangent to the rising part of the curve), wash-in area under the curve (WiAUC; area under the TIC from the time of arrival to the PE of arterial phase), half-decrease time (HDT; the time required to decrease from PE of arterial phase to 1/2 PE of arterial phase), fall time (FT; the time of contrast agent intensity from 95 to 5% PE of arterial phase), wash-out rate (WoR; the minimum slope of the curve, represented as a tangent at the descending part of the curve), wash-out area under the curve (WoAUC; the area under the TIC from the PE of arterial phase to the end of the curve), mean transit time (the time it takes for the intensity of the contrast agent to reach PE of arterial phase from 5% PE of arterial phase and then drop to 50% PE of arterial phase), and WiWoAUC (WiWoAUC + WiAUC, the area of the total curve) (Figs. 2 and 3).

### Statistical analysis

The mean and standard deviation of the age was calculated, and the distribution of ages and tumor diameters was analyzed. A univariate analysis and the chi-squared test or Fisher's exact test. Continuous variables used Student t-test or Mann–Whitney U-test when it’s appropriate. The specificity, sensitivity and positive predictive value, negative predictive value, and accuracy were calculated for the area under the curve (AUC). To determine the optimal cutoff value for DCE-US parameters, SPSS software was used to calculate the Youden index (Youden index = maxc {Sensitivity + Specificity − 1}) of all possible cutoff values (c). All data were calculated using the software program SPSS Statistics 22.0 (IBM, Armonk, USA). *P* value < 0.05 was considered statistically.

## Results

### Patient characteristics

Among the 120 patients in this study, there were 111 males (92.5%), with an average age of 55.2 ± 11.2 y (Fig. 1). Among the enrolled patients, there were 84 cases of high Ki-67 expression (> 10% immune-reactivity) HCC lesions and 36 cases of low Ki-67 expression (≤ 10% immunoreactivity) HCC lesions (see Table 1).

**Table 1:** The features of patients between high Ki-67 expression and low Ki-67 expressing group

Characteristic	High Ki-67 expression (*n* = 84)	Low Ki-67 expressing (*n* = 36)	*p*
*Clinicopathological* *features*			
Gender			
Male, *n* (%)	75	35	0.149
Age, mean ± SD, years	54.0 ± 11.4	57.8 ± 10.4	0.093
Cirrhosis, *n* (%)	56	28	0.224
HBsAg-positive, *n* (%)	69	31	0.593
AFP, ng/ml	2055.3 ± 7778.5	109.7 ± 292.9	0.137
CEA, ng/ml	2.7 ± 1.5	3.4 ± 2.3	0.087
CA19-9, ng/ml	53.0 ± 271.2	16.5 ± 12.3	0.487
CA125, ng/ml	14.1 ± 13.7	14.5 ± 7.2	0.935
Alanine aminotransferase, U/L	33.7 ± 25.9	31.0 ± 21.3	0.578
Aspartate aminotransferase, U/L	37.1 ± 35.2	28.2 ± 10.8	0.139
Total bilirubin, U/L	16.0 ± 26.2	13.0 ± 6.2	0.497
Edmondson-Steiner grade ( I-II, III-IV)	18:66	16:20	0.010
Microvascular invasion	23	3	0.020
*Imaging features*			
Location (right lobe, left lobe, caudal lobe)	56:27:1	22:13:1	0.734
Echo (high, low)	20:64	28:8	0.851
Tumor size (cm)	5.1 ± 2.9	4.5 ± 2.3	0.001
Blood flow signal (yes, no)	68:18	26:10	0.451
Inner diameter of main portal vein (cm)	1.2 ± 0.1	1.2 ± 0.2	0.770
*CEUS*			
Arterial phase (hype, iso, hypo-enhancement)	82:2:0	34:1:1	0.305
Portal phase (hype,iso,hypo-enhancement)	5:55:24	5:19:12	0.252
Delayed phase (hype,iso,hypo-enhancement)	0:8:76	0:5:31	0.481
Kupffer phase (hype,iso,hypo-enhancement)	0:1:83	0:1:35	0.534

*AFP* Alphafetoprotein; *CEA* Carcinoembryonic antigen; *CA125* Carbohydrate antigen125; *CA199* Carbohydrate antigen199; *CEUS* contrast-enhanced ultrasound

### Features predictors of Ki-67 expression

**Fig. 4:**
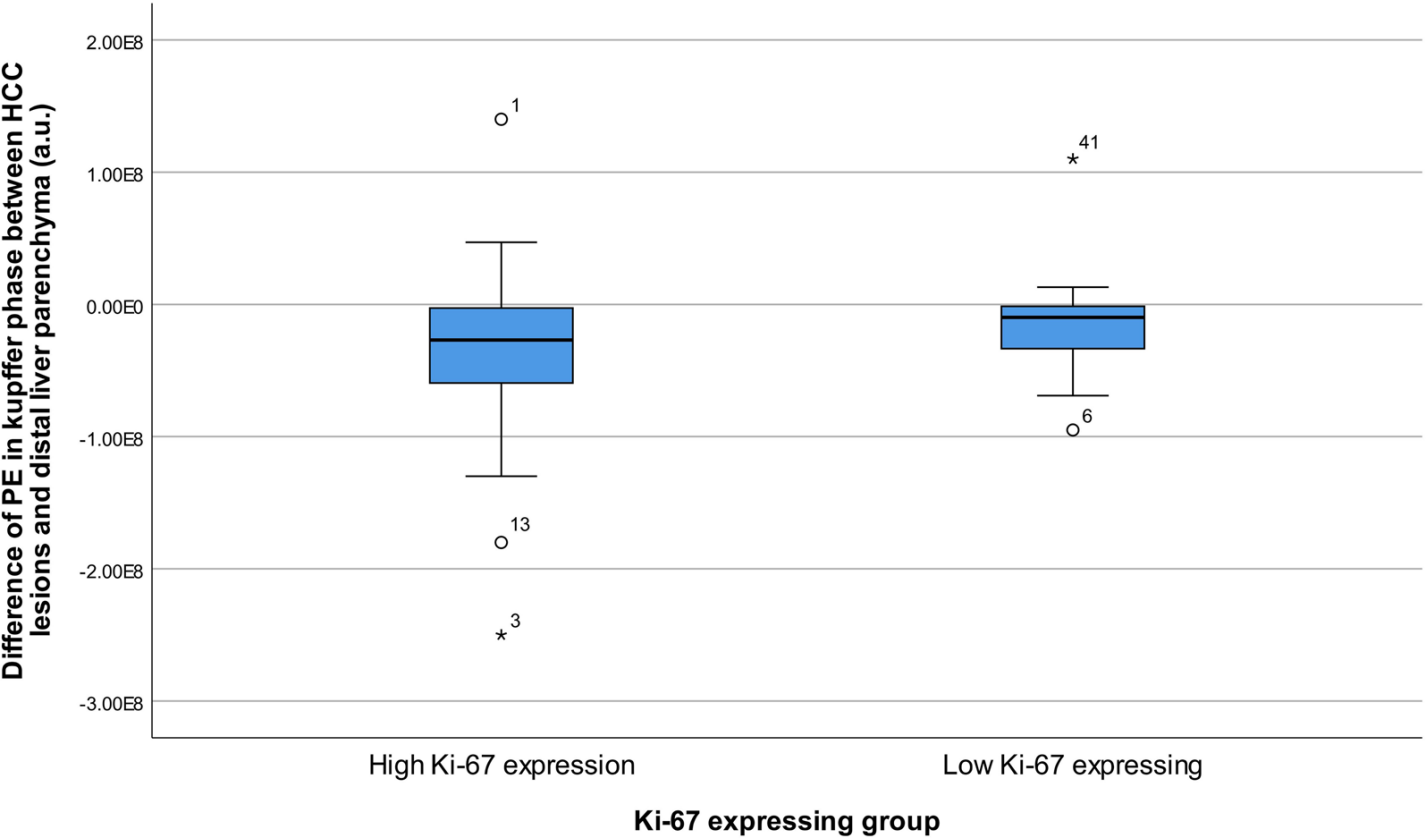
Dynamic contrast-enhanced ultrasound and quantitative parameters of hepatocellular carcinoma lesions. Among all dynamic contrast-enhanced ultrasound quantitative parameters, the difference of PE in the Kupffer phase between hepatocellular carcinoma lesions and distal liver parenchyma was higher in the low Ki-67 expression group than in the high Ki-67 expression group (*p* = 0.045). PE, peak energy

Clinicopathological features (pathological grade and microvascular invasion) were significantly different between the high Ki-67 expression and low Ki-67 expression groups (*p* = 0.010 and *p* = 0.020, respectively). Other clinicopathological and grayscale imaging factors, such as cirrhosis, sex, and tumor size, did not exhibit significant differences (*p* > 0.05) (Table 1). On CEUS, 116 (96.7%) HCCs lesions showed obvious hyper-enhancement in the arterial phase, 36 (30.0%) showed hypo-enhancement in the portal phase, 107 (89.2%) showed hypo-enhancement in the delayed phase, and 118 (98.3%) showed hypo-enhancement in the Kupffer phase. CEUS performance in the arterial, portal, delayed, and Kupffer phases did not exhibit significant differences between the high Ki-67 expression and low Ki-67 expression groups (*p* > 0.05). (Table 1) (Fig. 4).

In the high Ki-67 expression group, the DCE-US quantitative parameter FT and PE in the arterial phase were significantly different between the HCC lesions (21.9 ± 30.6 s, 1.3 × 10^8^ ± 1.0 × 10^8^ a.u, respectively) and the distal liver parenchyma (9.3 ± 36.5 s, 9.4 × 10^7^ ± 9.2 × 10^7^ a.u, respectively) (*p* = 0.016, *p* = 0.025, respectively). In the low Ki-67 expression group, FT and PE in the arterial phase showed no significant difference between the HCC lesions (16.7 ± 25.1 s, 1.1 × 10^8^ ± 9.7 × 10^7^ a.u, respectively) and the distal liver parenchyma (4.7 ± 34.9 s, 1.1 × 10^8^ ± 9.4 × 10^7^ a.u, respectively) (*p* = 0.104, *p* = 0.773, respectively). However, the DCE-US quantitative parameter PE in the Kupffer phase was significantly different between the HCC lesions (3.7 × 10^7^ ± 5.3 × 10^7^ a.u.) and the distal liver parenchyma (7.0 × 10^7^ ± 6.8 × 10^7^ a.u.) (*p* = 0.029). There was no significant difference between PE in the Kupffer phase of HCC lesions and PE in the Kupffer phase of the distal liver parenchyma in the high Ki-67 expression group (*p* = 0.650).

**Fig. 5:**
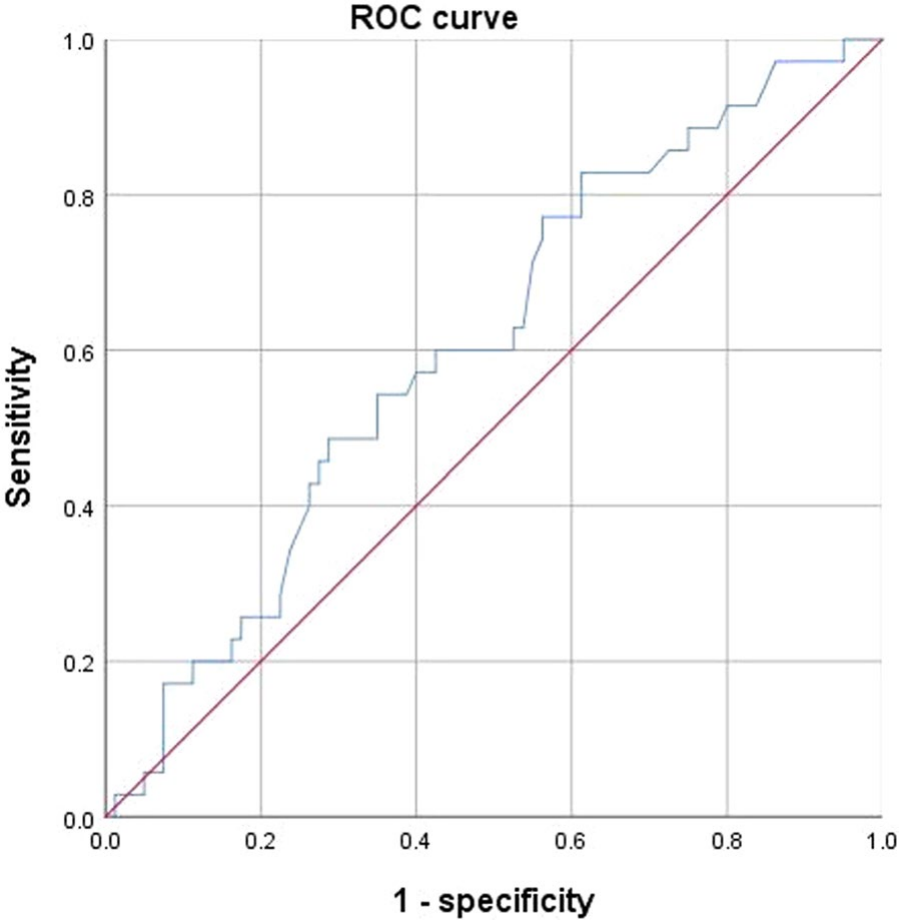
ROC curves of the difference of PE in the Kupffer phase between HCC lesions and distal liver parenchyma < − 40,000,000 a.u for predicting high Ki-67 expression group. ROC, receiver operating characteristic; PE, peak energy

Further quantitative analysis showed that when comparing the high Ki-67 expression and low Ki-67 expression groups, differences in PE in the Kupffer phase between HCC lesions and distal liver parenchyma were statistically different (*p* = 0.045). (Table 2)(Fig. 3). The difference in PE in the Kupffer phase between HCC lesions and distal liver parenchyma < − 40,000,000 a.u. may contribute to a more accurate diagnosis of the high Ki-67 expression group, and the sensitivity and specificity for distinguishing the high Ki-67 expression from the low Ki-67 expression group was 82.9% and 38.7%, respectively (Tables 3) (Fig. 5).

**Table 2:** The DCE-US features of patients between high Ki-67 expression and low Ki-67 expressing group

Characteristic	High Ki-67 expression (*n* = 84)	Low Ki-67 expressing (*n* = 36)	*p*
*DCE-US*			
HCC lesions			
CAT (s)	13.4 ± 17.4	14.9 ± 16.5	0.673
FT (s)	21.9 ± 30.0	16.7 ± 25.1	0.369
HDT (s)	74.6 ± 33.4	74.2 ± 38.7	0.955
PE in arterial phase (a.u.)	1.3 × 10^8^ ± 1.0 × 10^8^	1.1 × 10^8^ ± 9.7 × 10^7^	0.414
RT (s)	17.5 ± 26.5	22.5 ± 30.2	0.521
TTP (s)	30.6 ± 27.4	34.6 ± 28.6	0.762
WiAUC	1.2 × 10^9^ ± 1.3 × 10^9^	1.1 × 10^9^ ± 1.1 × 10^9^	0.819
WiR (a.u./s)	2.6 × 10^7^ ± 1.0 × 10^8^	2.4 × 10^8^ ± 1.2 × 10^9^	0.089
WiWoAUC	3.9 × 10^9^ ± 2.9 × 10^9^	3.2 × 10^9^ ± 4.3 × 10^9^	0.425
WoAUC	2.8 × 10^9^ ± 3.9 × 10^9^	2.1 × 10^9^ ± 3.6 × 10^9^	0.405
WoR (a.u./s)	− 6.3 × 10^6^ ± 8 × 10^6^	− 7.3 × 10^6^ ± 9.0 × 10^6^	0.593
mTT (s)	15.4 ± 16.4	14.9 ± 13.8	0.891
PE in Kupffer phase (a.u.)	3.5 × 10^7^ ± 4.4 × 10^7^	7.5 × 10^7^ ± 2.8 × 10^8^	0.224
*Difference between HCC lesions and distal liver parenchyma*			
CAT (s)	− 3.6 ± 20.7	− 3.2 ± 11.3	0.918
FT (s)	16.0 ± 34.7	17.1 ± 39.5	0.879
HDT (s)	− 2.1 ± 28.4	0.4 ± 14.4	0.630
PE in arterial phase (a.u.)	2.4 × 10^7^ ± 8.8 × 10^7^	2.5 × 10^6^ ± 8.1 × 10^7^	0.208
RT (s)	− 10.3 ± 30.9	− 9.2 ± 30.8	0.857
TTP (s)	− 13.7 ± 31.3	− 12.1 ± 27.8	0.791
WiAUC	− 2.3 × 10^5^ ± 1.2 × 10^5^	− 9.8 × 10^5^ ± 5.0 × 10^7^	0.111
WiR (a.u./s)	1.8 × 10^6^ ± 2.2 × 10^7^	4.6 × 10^5^ ± 1.2 × 10^7^	0.738
WiWoAUC	1.0 × 10^9^ ± 5.2 × 10^9^	− 7.2 × 10^8^ ± 4.3 × 10^9^	0.082
WoAUC	1.3 × 10^9^ ± 4.1 × 10^9^	2.3 × 10^8^ ± 3.5 × 10^9^	0.179
WoR (a.u./s)	− 7.6 × 10^7^ ± 6.9 × 10^8^	− 5.4 × 10^7^ ± 3.2 × 10^6^	0.124
mTT (s)	− 0.4 ± 23.2	− 0.1 ± 16.4	0.939
PE in Kupffer phase (a.u.)	− 3.4 × 10^6^ ± 4.8 × 10^7^	− 1.8 × 10^7^ ± 3.4 × 10^7^	0.045

*CAT* contrast agent arrival time; *PE* peak energy; *TTP* time to peak; *RT* rise time; *WiR* wash-in area under the curve; *WiAUC* wash-in area under the curve; *HDT* half decrease time; *FT* fall time; *WoR* wash-out rate; *WoAUC* wash-out area under the curve; *mTT* mean transit time; *WiWoAUC* WiWoAUC + WiAUC

**Table 3:** Diagnostic performance of variables

Variables	Cutoff value	AUC	Sensitivity	Specificity	Positive predictive value	Negative predictive value	Accuracy
Pathological grade (poorly differentiated)		0.627	0.786 [0.680, 0.865]	0.444 [0.283, 0.617]	0.767 [0.662, 0.849]	0.471 [0.302, 0.646]	0.683 [0.691, 0.881]
Microvascular invasion		0.581	0.274 [0.185, 0.384]	0.889 [0.730, 0.964]	0.852 [0.654, 0.951]	0.344 [0.251, 0.450]	0.458 [0.291, 0.636]
Difference of PE in Kupffer phase between HCC lesions and distal liver parenchyma (a.u.)	− 40,000,000	0.607	0.829 [0.733, 0.903]	0.387 [0.236, 0.565]	0.761 [0.659, 0.841]	0.500 [0.311, 0.689]	0.700 [0.564, 0.836]

*PE* peak energy; *AUC* area under the curve

## Discussion

**Fig. 6:**
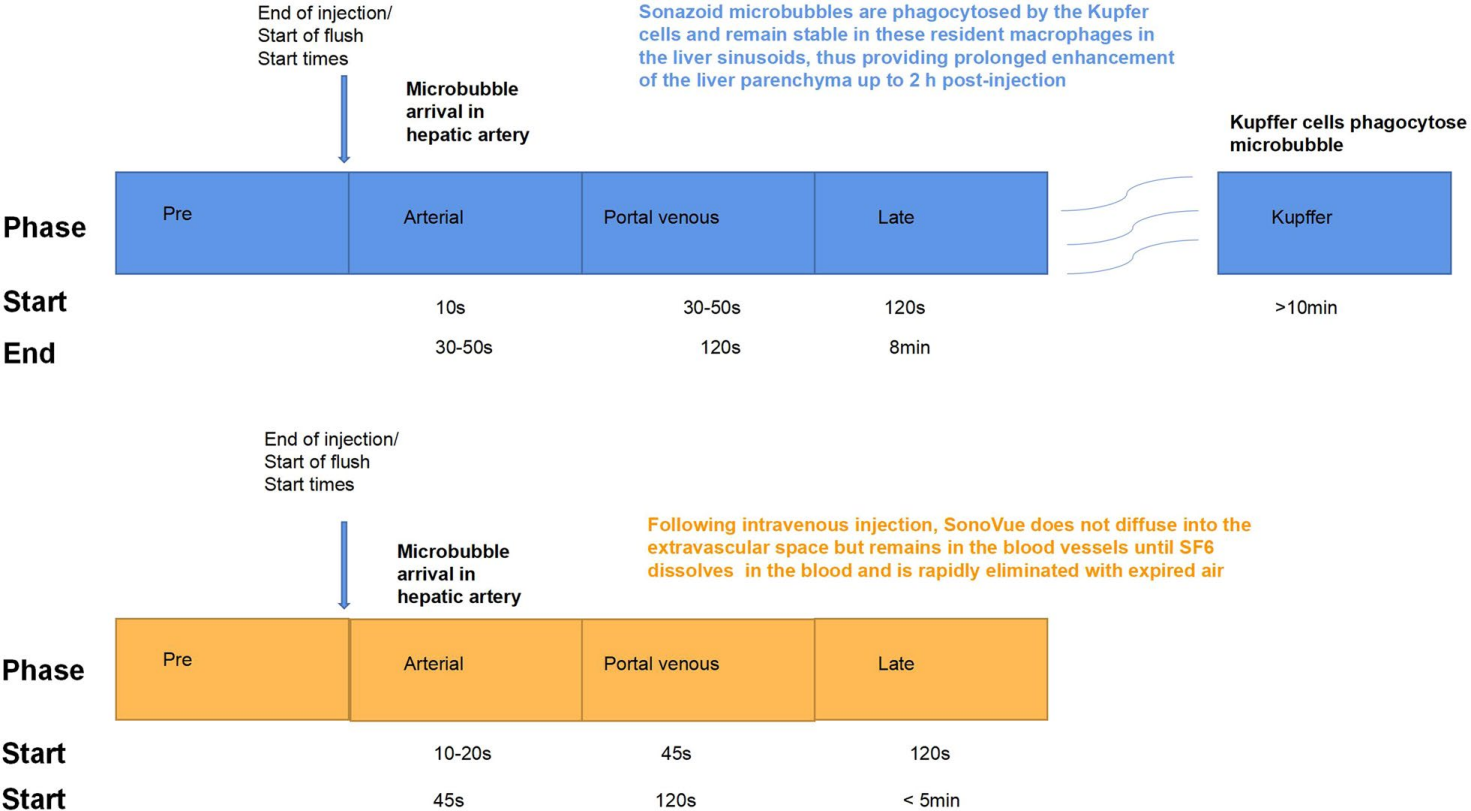
The differences between second generation contrast media in which hepatic artery and von Kupffer cells

The proliferation state of tumor cells is an important parameter that reflects their biological characteristics and directly affects tumor prognosis and treatment efficiency. As a biomarker with high sensitivity and specificity, Ki-67 is widely used as a proliferative and prognostic factor in HCC. Our study found that FT and PE in the arterial phase were significantly different between the HCC lesions and the distal liver parenchyma in the high Ki-67 expression group, especially the difference in PE in the Kupffer phase between HCC lesions and distal liver parenchyma < − 4.0 × 10^7^ a.u. may contribute to a more accurate diagnosis in the high Ki-67 expression group (Fig. 6).

High Ki-67 expression of HCC indicated the potential deterioration of HCC, as presented by poor histological differentiation and more microvascular invasion. High Ki-67 LI group tended to show higher PKM2 mRNA expression and FDG uptake than low or intermediate LI groups. Regulation of glycolysis via GLUT-1 and PKM2 may influence the proliferative activity of HCC to a certain extent [19].

Our study found that FT and PE in the arterial phase were significantly different between HCC lesions and the distal liver parenchyma in the high Ki-67 expression group, but these differences were not observed in the low Ki-67 expression group. Since high Ki-67 expression represents an active state of cell proliferation, new blood vessels may be required to promote tumor growth. Sonazoid CEUS-based arterial phase images can best reveal information about tumor neovascularization.

In the ROC analysis, the difference in PE in the Kupffer phase between HCC lesions and distal liver parenchyma had a high ability to recognize Ki-67 expression. The Kupffer phase enhancement is due to the extravasation of the Sonazoid contrast agent and subsequent uptake by Kupffer cells [20]. The proportion of Kupffer cells is related to the histological grade or degree of differentiation of liver malignancies [21]. The perfusion defects seen on Kupffer imaging and the degree of histological malignancy of HCC are correlated. That is because as HCC progresses, Kupffer cells may become fewer or even disappear, leading to an area clear of contrast material or a perfusion defect in Kupffer imaging [22]. Therefore, PE in the Kupffer phase is related to the histological grade. Since Ki-67 is correlated to cellular proliferation, the high Ki-67 group tended to poorly differentiated than the low Ki-67 group. This may indicate that in the high Ki-67 group, the difference in PE in the Kupffer phase between the lesions and the surrounding liver tissue is small.

Presently, there is no unified standard for the differentiation of the cutoff value of high and low expression groups of Ki-67. A cutoff value of 10% for Ki-67 expression was used in our study, whereas a cutoff value of 14% was used in other studies [23]. In this case, high Ki-67 expression was closely correlated with poor differentiation (correlation coefficient − 0.238, *p* = 009). The results of this study are consistent with those of previous studies [2].

This study had several limitations. First, the study sample size was small, especially in the low Ki-67 expression group. Second, our study participants were recruited from a single institution, which limits the generalizability of our findings to other institutions or settings. Last, CEUS is an operator-dependent technique, and all the exams were performed by a single radiologist, which may limit the generalizability of our findings to other radiologists.

In summary, our study suggests that dynamic contrast-enhanced ultrasound parameters have the potential as biomarkers for predicting Ki-67 status in patients with HCC.

## Data Availability

The dataset used or analyzed during the current study are available from the corresponding author on reasonable request.

## Abbreviations

A.U: Absorbance unit
CEUS: Contrast-enhanced ultrasound
DCE-US: Dynamic contrast-enhanced ultrasound
FT: Fall time
HCC: Hepatocellular carcinoma
PE: Peak energy
